# Systematic review on the recurrence of postoperative nausea and vomiting after a first episode in the recovery room – implications for the treatment of PONV and related clinical trials

**DOI:** 10.1186/1471-2253-6-14

**Published:** 2006-12-13

**Authors:** Leopold HJ Eberhart, Silke Frank, Henning Lange, Astrid M Morin, André Scherag, Hinnerk Wulf, Peter Kranke

**Affiliations:** 1 Department of Anaesthesiology and Critical Care Medicine, Philipps-University Marburg, Germany; 2 Medical University Library, Philipps-University Marburg, Germany; 3 Institute of Medical Biometry and Epidemiology, Philipps-University Marburg, Germany; 4 Department of Anaesthesiology, University of Würzburg, Germany

## Abstract

**Background:**

Despite the presence of a plethora of publications on the prevention of postoperative nausea and vomiting (PONV) only little is known how to treat established symptoms. Besides the high effort of performing these efficacy trials (much more patients must give their consent than are actually included in a study) and ethical concerns, little is known about the rate of re-occurring PONV/vomiting after placebo. As a consequence investigators will have difficulties defining a clinically relevant effect for the new treatment which is crucial for any planning. A quantitative systematic review was performed in order to provide more reliable estimates of the incidence of re-occurring PONV/vomiting after placebo and to help investigators defining a clinically relevant treatment effect.

**Methods:**

A systematic search of the literature was performed using an extended search strategy of a previous review. Data on the recurrence of PONV (any nausea or emetic symptom) and vomiting (retching or vomiting) was extracted from published reports treating PONV with placebo and unpublished results from two observational trials where no treatment was given. A nonlinear random effects model was used to calculate estimates of the recurrence of symptoms and their 95%-confidence intervals (95%-CI).

**Results:**

A total of 29 trials (including the unpublished data) were eligible for the calculations. Depending on the length of observation after administering placebo or no treatment the recurrence rate of PONV was between 65% (95%-CI: 53%...75%) and 84% (95%-CI: 73%...91%) and that of vomiting was between 65% (95%-CI: 44%...81%) and 78% (95%-CI: 59%...90%).

**Conclusion:**

Almost all trials showed a considerable and consistently high rate of recurrence of emetic symptoms after placebo highlighting the need for a consequent antiemetic treatment. Future (placebo) controlled efficacy trials may use the presented empirical estimates for defining clinically relevant effects and for statistical power considerations.

## Background

Postoperative nausea and vomiting (PONV) are frequent adverse events after anaesthesia. In western countries about 10% of the population undergoes a surgical procedure every year. Assuming that still a quarter of all patients suffer from PONV, more than 18 million patients are nauseated or vomit after their surgery in Europe. Although these symptoms are self-limiting and rarely cause major medical problems they are distressing for the patient and may have negative economic implications, e.g. by causing unanticipated hospital admission in surgical outpatients due to intractable vomiting.

There is some evidence that patients at a considerable high risk for developing PONV can benefit from prophylactic antiemetic treatment [1]. However, applying the same approach for patients at a low or moderate risk is not unequivocally supported and fails to improve patient satisfaction compared to symptomatic treatment in the postoperative recovery room [1]. The main reason for the poor results of any conventional antiemetic strategy is that the number of patients that need to be treated prophylactically highly depends on the baseline risk of the population. For instance, if high risk patients with an expected risk for PONV of 80% are treated with an antiemetic intervention that halves this baseline risk then the achieved absolute risk reduction of 40% translates into a NNT of 2.5 that without doubt is highly efficient. In a population with a low risk for PONV (e.g. 10%), however, the same intervention will lead to a (disappointing) 5% absolute risk reduction and the NNT is 20.

This simple calculation demonstrates that routine antiemetic prophylaxis is not indicated from a medical (potential side effects of antiemetics) and economic point of view (increased costs). However, the optimal threshold (percentage of expected risk) at which prophylaxis is indicated cannot be quantified since several other issues modify the perceived risk. Thus, a recent consensus conference did not give a clear recommendation regarding this point [2].

The situation is further complicated by the lack of a clinically satisfying solution to predict PONV. The accuracy of prediction using one of the available simple scoring systems [3,4] is at best increased to a discriminating power of about 70% compared to a random guess with a pre-hoc accuracy of 50% [5,6]. Furthermore, even if there was a perfect prognostic tool, theoretical consideration using computer simulation of the effectiveness and efficacy of various prophylactic antiemetic strategies could not identify one optimal approach since the "most effective" or "most efficient" strategy strongly depends on the distribution of patients in a given institution [7]. As a consequence, clinical strategies to deal with PONV in the postanaesthetic care unit vary widely between different institutions [8].

An alternative to administering prophylactic antiemetics is to treat established nausea and vomiting postoperatively. This strategy has been shown to be more cost-effective than prophylaxis [9]. However, a systematic review evaluating studies on treatment of PONV stated that there is a discrepancy between the plethora of trials on prevention of PONV and the paucity of trials on the treatment of established symptoms [10]. There are several reasons for this obvious disparity. First, manufacturers of antiemetic drugs have more commercial interest in prophylaxis strategies which may be the reason for the complete lack of trials with older classic antiemetics when treatment of established PONV is concerned. Second, in the absence of evidence in favour of a (gold) standard for treatment of established PONV, one will have to utilize placebo control groups. Since ethics committees have the duty to balance between the patients' interest and the scientific benefit, it can be speculated that they will be reluctant to approve such trials since there is good evidence from several surveys of in- and outpatients [11,12] that postoperative nausea and vomiting is as unpleasant as postoperative pain. The problems related to placebo treatment are supported by thorough criticism of trialists and sponsoring companies performing placebo-controlled trials in the prophylaxis of postoperative nausea and vomiting [13,14] – trials that all provided sufficient antiemetic rescue medications for all participants! Third, and most importantly, therapeutic trials are more difficult to perform. Of the total number of patients giving informed consent to participate in such a trial, only about 20–30% (at "best") can be expected to be randomized due to the fact that the majority of patients never suffer from PONV. In addition, the number of patients that are willing to participate in a placebo-controlled PONV-trial will probably be rather low, especially in patients with a high risk to develop emetic symptoms, e.g. due to previous PONV. Furthermore, study protocols will have to be very precise concerning the inclusion criteria and the proceedings in case of adverse events (e.g. because of the necessary definitions when to administer a rescue antiemetic, etc.) and these regulations will probably result in artificial circumstances that might not be representative of a "real world" situation and thus such trials might lack external validity.

Besides all these obstacles, there is an obvious need for trials showing efficacy of an antiemetic for the treatment of PONV-symptoms prior to its routine application. In the absence of an accepted (gold) standard treatment [15], one may have to use placebo control treatment arms. Any efficacy consideration in terms of clinically relevant treatment effects, however, implies some knowledge about the incidence of an event with placebo treatment. Confronted with this dilemma we decided to update a recent review on trials on the efficacy of therapeutic antiemetic interventions [10]. This material was amended by unpublished data that were collected in order to validate a variety of different risk scores to predict the occurrence of PONV [5] and to train and validate an artificial neural network for the same purpose [16].

Thus, the aim of this study was twofold. First, it was set up to provide a more reliable incidence estimate for the recurrence of emetic symptoms following an initial emetic episode and after receiving placebo during the early postoperative period (e.g. in the recovery room). Secondly, these data were to propose the choice of clinically relevant effects for future trials studying antiemetic interventions for the treatment of PONV. Based on these data we will also discuss the possibility to perform PONV treatment trials with no placebo comparator group by using a virtual benchmark for such trials.

## Methods

In order to determine a more reliable incidence estimate for patients with reappearance of nausea or vomiting after a first episode of these symptoms in the recovery room, a systematic review of trials was performed that documented the rate of recurrence of PONV after giving placebo or no treatment. For data retrieval and extraction we updated a previous systematic review of Kazemi-Kjellberg et al. published in 2000 [10].

We used the search strategies of this systematic review and created modified algorithms with the help of a librarian (S.F.). We used the following search terms or roots for inclusion "postoperative OR postsurg*", "nausea OR vomit* OR emesis OR emetic OR retch*", "treatment OR control" and the phrases "chemotherapy OR radiotherapy OR prophylaxis or prevention" for exclusion. There was no restriction to placebo-controlled trials. Given this strategy, we searched Medline, Old Medline (reaching back to 1950 – 1959 Current List of Medical Literature), EMBASE, and the Cochrane Controlled Trials register (CENTRAL) aiming at all trials that provide numbers about the incidence of patients that suffer from any emetic sequelae after a first episode of PONV during the early postoperative period. The date of the last literature search was 16^th ^December 2005. Additionally we hand-searched the references of all matching papers and review articles for additional reports but did not contact the pharmaceutical industry for further unpublished data. In contrary to the first systematic review [10] we did not exclude articles that reported the incidence of PONV instead of two distinct incidences for nausea and vomiting. For the given study, these PONV incidences were equated with nausea since results from almost all trials suggests that vomiting without prodromal nausea is extremely rare. Thus, "PONV" was defined as any nausea or vomiting or retching (with or without a rescue medication at this stage) in the postoperative period and "vomiting" as retching or vomiting postoperatively. Most of the identified trials did not use an intraoperative antiemetic prophylaxis and do not report risk factors for PONV present in the patients who then suffered from nausea or vomiting in the recovery room. However, studies were not excluded in case such explicit information were lacking. The data from the identified reports were independently extracted by two researchers. The Oxford-score (Jadad-score) was assessed for each study [17]. In the case of disagreement between the two results a referee (L.E.) was consulted to solve the disagreement. The analysis was performed according to the recommendations of the QUORUM-statement [18].

### Re-analyses of two observational trials

Results of two observational surveys on the occurrence of PONV [5,16] were re-analyzed. Both studies covering a total of 3608 patients had been approved by the local ethics committee and informed consent was obtained from each patient. Both trials were designed to evaluate potential risk factors for PONV. Due to the observational character of the surveys, the type and the length of surgery and anaesthesia were recorded but no efforts were made to modify the drugs or techniques used. Both were chosen according to local standards at the discretion of the attending anaesthetist. Of all the investigated patients only those presenting nausea and/or vomiting in the recovery room were analyzed for the rate of recurrence of these symptoms within the first 24 hours after emergence from anaesthesia. Nausea was defined as an active complaint of the patient that lasted at least 5 minutes. Retching was rated as vomiting (emetic episode). Patients with these symptoms were treated at the discretion of the attending nursing staff. Again, antiemetic therapy was not standardized and comprised mainly of dopamine antagonists (metoclopramide and droperidol) and to a lower extent also antihistamines (e.g. dimenhydrinate), since newer antiemetics like 5-HT_3_-antagonists were not available for postoperative treatment at that time the data were recorded.

After discharge from the post-anaesthesia care unit (PACU), patients were visited on the ward 6–8 hours and 24–26 hours postoperatively by a specially trained observer. Both, the patients and the nursing staff, were asked whether nausea and/or an emetic episode had occurred. Additionally, the medical records were screened in order not to miss symptoms of PONV in a patient.

### Statistical analysis

It is evident that the incidence of PONV is highly dependent on the observational interval [19]. As the different studies applied heterogeneous follow-up periods for ascertaining PONV and vomiting, it was decided to do an a priori classification of the studies investigating adults (0–1 h, 0–2 h, 0–6 h, and 0–24 h of postoperative follow-up). For each follow-up group separate pooled incidence estimates were obtained. Note that single studies may appear up to four times in different groups i.e. one study may have presented data for two follow-up intervals for both PONV and vomiting (see details in table 1). Furthermore, the number of studies within each group can be considerably small. In order to deal with this problem, a nonlinear random effects model (NLMIXED in SAS Version 8.02) was applied to obtain pooled incidence estimates and the respective 95% confidence intervals as recommended by Kuss [20]. The analyses were done twice including/excluding the data from the two observational trials to investigate whether the observational character of these studies had an effect on the estimates. For simple interval estimates of rates the classical method of Clopper and Pearson [21] was applied.

**Table 1:** Success rates of placebo or no treatment after a first emetic episode postoperatively.

Reference	Oxford-scale (R/B/D)	type of surgery/patients	entry criteria	recurrent PONV after ... hours	n/N	recurrent vomiting after ... hours	n/N	recurrent PONV after ... hours	n/N	recurrent vomiting after ... hours	n/N
Alon, 1998 [28]	2/2/1	various	patients, experiencing nausea lasting > 10 min and/or emesis within 2 h after recovery from general anaesthesia	4	37/77	4	37/77	24	51/77	24	55/77
Anderson, 2004 [29]	1/1/0	various, outpatients	patients, spontaneously reporting nausea in the PACU	2	6/12						
Barton, 1975 [30]	2/1/0	various	patients, developing nausea/vomiting in the recovery period	1	15/25	1	12/26	3*	24/30	3*	21/30
Bodner, 1991 [31]	1/1/0	laparoscopy, female outpatients	patients, complaining of persisting nausea (lasting >10 min) and/or experienced at least 2 episodes of emesis/retching			2	33/36				
Boghaert, 1980 [32]	1/1/1	various	vomiting postoperatively			1	33/44			6	37/44
Bonica, 1958 [33]	1/1/1	various				0.5	186/272				
Borgeat, 1992 [34]	1/1/0	various	patients, exhibiting major or severe nausea with vomiting	0.02	17/26			0,5	19/26		
Diemunsch, 1997 [35]	1/1/0	various, primary gynaecological surgery	reporting nausea lasting 10 min or one emetic episode within 2 h in PACU	8	63/71					24	63/71
Diemunsch, 1999 [36]	1/1/0	abdominal or vaginal oophrectomy	experience of nausea and/or vomiting within 6 h of surgical recovery	6	16/18	6	14/18	24	18/18	24	17/18
Du Pen, 1992 [37]	1/1/1	primary gynaecological, outpatients	experience of nausea and/or vomiting within 2 h in the PACU	2	90/129			24	110/129		
Fragen, 1978 [38]	2/0/1	gynaecologic surgical procedures	retching or vomiting in the immediate postoperative period	2	26/30	2	18/30	4	29/30	4	29/30
Fujii, 2004 [26]	2/2/1	laparoscopic cholecystectomy	experience of nausea lasting more than 10 min or retching or vomiting within 3 h after recovery					24	10/20	24	4/20
Gan, 1999 [39]		ambulatory surgery	significant nausea or vomiting within 1 h of arrival in the recovery room	2	18/23	2	13/23				
Harper, 1998 [40]	2/2/1	laparoscopic gynaecological surgery	complaining of nausea and/or vomiting and requesting antiemetic treatment in the recovery room			1,5	2/12	3	9/12	3	6/12
Kauste, 1986 [41]	1/1/0	elective orthopaedic surgery	complains of nausea or retched or vomiting of any severity			6	22/36	24	30/36	24	24/36
Khalil, 1996 [42]	2/2/1	paediatric surgery, outpatients	experience of 2 emetic episodes within 2 h of discontinuation of nitrous oxygen			2	120/183			24	149/179
Korttila, 1979 [43]	1/1/0	orthopaedic surgery	complains of nausea or retched or vomitinf of any severity					24	18/40		
Kovac, 1997 [44]	2/1/0	various, outpatients	postoperative nausea or vomiting within 2 h of arrival in the PACU			2	88/121	24	115/121	24	108/121
Kovac, 1999 [45]	2/1/1	various, outpatients	experience of PONV or requesting antiemetic therapy within 2 h of the end of anaesthesia	2	122/214			24	145/214		
Larijani, 1991 [46]	2/1/0	orthopaedic/gynaecologic surgery	complaining of nausea or having a vomiting episode within 2 h of arrival in the recovery room	4	13/18	4	13/18				
Lobera, 1981 [47]	1/1/0	breast surgery	presenting nausea and/or vomiting	0.33	38/60						
Loeser, 1979 [48]	1/1/0	n/a	one or more episodes of vomiting in the recovery room			2	11/16				
Polati, 1997 [49]	2/2/1	gynaecologic surgery	experience of persistent nausea with at least one emetic episode within 4 h of recovery	1	39/60			48	53/60		
Rung, 1997 [50]	2/2/1	orthopaedic/gynaecologic surgery	experience of nausea and/or emesis and request of an antiemetic at any time after the start of opioid administration	6	27/32			24	27/32		
Scuderi, 1993 [51]	2/1/0	outpatients	if symptomatic treatment for persistent nausea or vomiting was necessary			2*	26/55				
Stockman, 1978 [52]	1/1/0	various	patients, who had a sufficient degree of postoperative nausea and/or vomiting to warrant antiemetic therapy			1	12/21				
Taylor, 1997 [53]	2/1/0	gynaecological surgery	patients, who developed PONV within 4 hours of the end of surgery	6	111/133	6	98/133	24	116/133	24	107/133
van Leeuwen, 1980 [54]	1/1/0	general surgery, urology, plastic & vascular	vomiting postoperatively			6	30/48				
Zegveld, 1978 [55]	1/1/1	abdominal and others	after vomiting had occurred	6	34/58						
Eberhart, unpublished data [5,16]	n/a	various	Any nausea, retching or vomiting for at least 5 minutes	2,2	128/204			22,5	175/204		

* time intervals were not exactly defined in the original paper and were judged according to additional information in the text, e.g. data on the length of stay in the recovery room, discharge times, etc.

## Results

### Systematic review of the literature

Using several search strategies we identified 163 potentially relevant papers. After reviewing the abstracts of these reports by two independent researchers (L.E. and P.K.) 63 articles were intensively screened as full texts. Of these 35 had to be excluded for the following reasons:

• lack of a placebo or no treatment group: n = 27

• overt double publication (see reference [10] for details): n = 3

• prophylaxis and treatment not separable: n = 2

• studies performed in paediatric patients n = 2

• no information on PONV or vomiting provided (only need for antiemetics): n = 1

Finally, within the remaining 28 publications, placebo or no treatment was used in at least some of the patients presenting with emetic symptoms in the early postoperative period and were used for the calculation of the pooled incidences of PONV and vomiting reappearance. Of these, ten trials had an Oxford-score of 4 or 5 (out of 5 possible). The lowest score was 2 the greatest 5 with a median of 3. All identified studies had at least one active comparator group (tropisetron, ondansetron, dolasetron, granisetron, propofol, haloperidol, droperidol, domperidone, metoclopramide, alizapride, tiapride, and isopropyl alcohol for inhalation). All trails were placebo controlled. The main findings of the extracted papers are listed in table 1.

The unpublished results of the two observational trials [5,16] are listed at the end of table 1. In the latter a total of 3608 adult patients were observed prospectively. During the stay in the recovery room (mean duration of stay: 116 minutes) 584 (16%) of them developed nausea or vomiting. Of these 380 patients were immediately treated with a broad variety of antiemetic interventions. The remaining 204 patients (35%) did not receive an antiemetic drug. Reasons for the decision not to administer an antiemetic was spontaneous relief of symptoms in most of the cases (e.g. after an episode of vomiting) or presence of contraindications against antiemetic drugs that were commonly used during the time the study was performed. Of the 204 patients who did not receive an antiemetic after a first episode of PONV, 128 (63%) developed further emetic sequelae during their stay at the recovery room and were subsequently treated with an antiemetic. Of the remaining 76 patients discharged to the ward without antiemetic treatment, 47 suffered from PONV within the 24 hours observation period. Thus a total of 175 (= 128+47) of the 204 patients (86%; 95%-CI: 80%...90%) had reappearance of emetic symptoms after not receiving no treatment. Their data was included in the pooled analysis of the 0–24 hour period.

**Figure 1:**
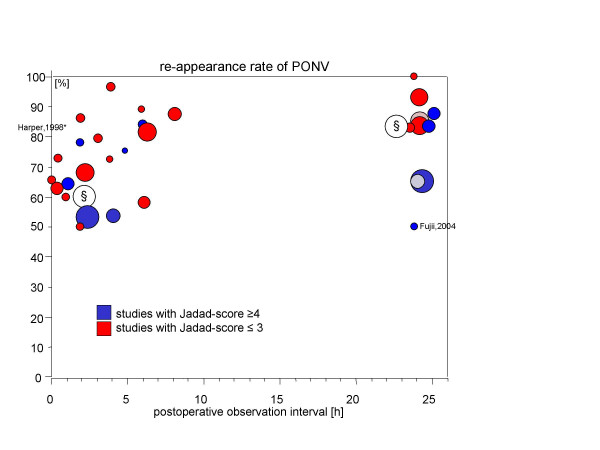
Rates of recurrent PONV are given on the y-axis depending on the length of the observation period (x-axis). The area of the circles represent the number of patients included in the trial. Furthermore, light shading indicates studies with an Oxford (Jadad) score ≤ 3 whereas dark shading indicates a Jadad score of ≥ 4. The unpublished results from two observational surveys are marked with "§".

**Figure 2:**
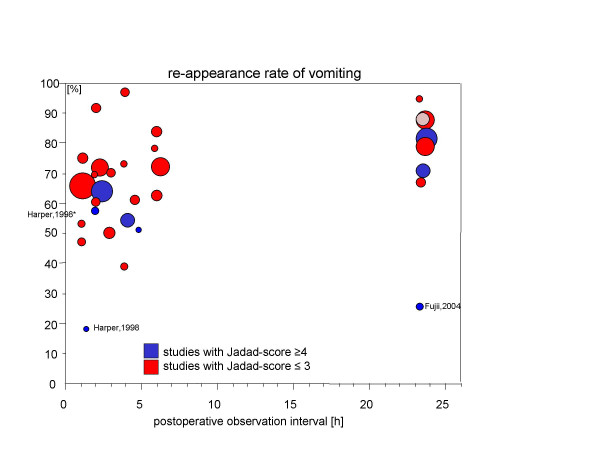
Rates of recurrent vomiting are given on the y-axis depending on the length of the observation period (x-axis). The area of the circles represent the number of patients included in the trial. Furthermore, light shading indicates studies with an Oxford (Jadad) score ≤ 3 whereas dark shading indicates a Jadad score of ≥ 4.

Figures 1 and 2 display the rate of recurrence of PONV and vomiting respectively as reported by the original trials (y-axis) depending on the time period of the observations (x-axis). The number of patients investigated in these trials is indicated by the size of the circular area whereas the "quality" of the reporting (Jadad-score) is coded by shading.

**Figure 3:**
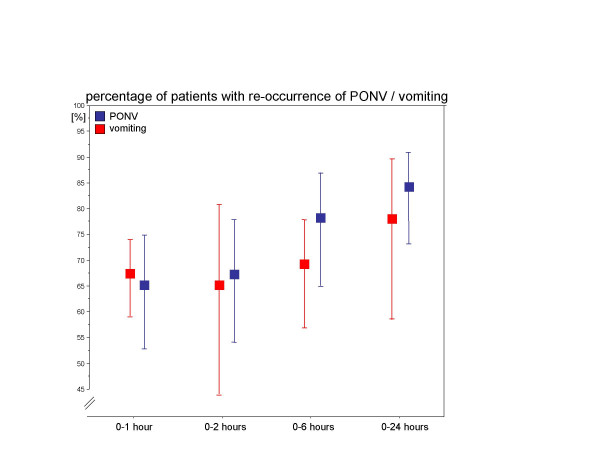
The pooled rate estimates and their 95%-confidence intervals for the recurrence of PONV (dark shading) and vomiting (light shading) within the different observation intervals.

The pooled estimates of the rate of recurrence after placebo comprise data from 28 trials. Depending on the observation interval and the outcome criteria (PONV versus vomiting – regardless whether treated or not treated at this stage) data of between 4 and 10 reports were used for an analysis (table 2, figure 3). As expected, except for the results of the 0–1 hour interval, an increasing incidence of PONV-sequelae for both the pooled PONV and vomiting estimates can be observed. Furthermore, the incidence of PONV which comprises nausea *and *vomiting is higher than the incidence of vomiting alone. However, the respective 95% confidence intervals are rather wide ranging between 51% and 92% for PONV and between 44% and 90% for vomiting (irrespective of the time interval). This is a result of the limited number of studies and the lack of trials with large sample size within each investigated time interval.

**Table 2:** The pooled estimates and their 95%-confidence intervals of the recurrence of PONV or vomiting.

Observation interval	Number of included studies*	Incidence of recurrence of ...	Pooled incidence (95%-confidence interval) [%]
0–1 hour	4 ^30,34,47,49^	PONV	65 (53...75)
	4 ^30,32,33,52^	vomiting	67 (59...74)
0–2 hours	7 ^§^	PONV	67 (54...78)
	5 ^29,37–39,45^	PONV	69 (51...83)
	8^31,38–40,42,44,48,51^	vomiting	65 (44...81)
0–6 hours	9^28,30,36,38,40,46,50,51,55^	PONV	79 (66...88)
	10 ^28,30,32,36,38,40,41,46,^	vomiting	70 (58...79)
	^53,54^		
0–24 hours	13 ^§^	PONV	84 (73...91)
	11 ^26,28,36,37,41,43–45,49,^	PONV	84 (71...92)
	^50,53^	vomiting	79 (61...90)
	8 ^26,28,35,36,41,42,44,53^		

* Please note that even if the absolute number of included trials is equal not necessarily the same trials were analyzed for the outcomes "PONV" on the one hand and "vomiting" on the other hand.

§ Pooled results using the random effects model with including the results of the two unpublished trials [5,16].

If, despite this variability, one wants to give recommendations for clinically relevant effects that can be considered of interest for antiemetic trials studying the treatment of PONV, one may consider two scenarios using the lower and the upper bound of the 95%-confidence interval of the pooled incidence of PONV recurrence. One of these scenarios, the "best-case" scenario is of special interest. It assumes a low rate of recurrent PONV after placebo/no treatment. Taking the 24-hour pooled estimates of patients suffering from an initial episode of PONV as an example, this means that at least about 73% of the patients can be expected to show recurrence of PONV. In this case, one may argue that the incidence of PONV recurrence within the first 24 hours under any investigational treatment of interest should be at least lower than 70%.

## Discussion

As pointed out in the introduction the treatment of established symptoms will remain a cornerstone of any strategy aiming to decrease the sequelae for patients resulting from postoperative nausea and vomiting despite the increasing use of prediction models and guidelines for PONV-prophylaxis. In short the main reasons are listed as follows:

- Prediction models for PONV for an individual patient that are applicable in the clinical practice have by now not turned out to be satisfactory [5,6]. Although the accuracy can be increased to some extent using sophisticated statistical methods [16], the main and limiting factor is the absence of strong predictive parameters [22].

- A clinical decision whether or not to administer an antiemetic measure is highly depended on the individual characteristics of the patient and the clinical circumstances. For instance, in the outpatient setting antiemetic prophylaxis is more often warranted than in inpatients. As a consequence, guidelines did not and will probably never define an exact threshold when antiemetic prophylaxis is indicated [2].

- Computer simulations on the effectiveness and efficacy of various antiemetic strategies have yielded no perfect solution. Instead, it is suggested to develop individualized standards at each institution depending on the composition of patient population [23].

The latter analysis further suggests that despite a liberal antiemetic prophylaxis PONV will remain a "10–20%-problem" in the postoperative period. Thus, studies on the treatment of PONV are urgently warranted. However, despite the need for such treatment studies it is very unlikely that such trials – especially placebo controlled trials – will be performed in the near future. Several reasons have been described in the introduction section.

There are only two realistic solutions to deal with this lack of sufficient data on antiemetic treatment.

One is that clinicians rely on the data that is available for the prophylaxis of PONV and extrapolate these results to the demands of treatment.

The other one is to fill this gap of evidence with data from well conducted clinical trials. In the absence of a well established (gold) standard treatment [15], one may stick to the need of running placebo-controlled trials despite their inherent difficulties. For this approach the calculated estimate for the recurring incidence of PONV can be helpful to plan such a placebo-controlled study. For example, using the 0–24 hour estimate of 84% (rate of recurring PONV) and assuming a relative risk reduction by a certain antiemetic treatment then the expected incidence in the active treatment group would be 59% (= 0.84. [1-0.3]). This absolute risk reduction of 25% points is a reasonable margin for a clinically relevant effect and a sample size of 2 × 57 patients can be determined for two parallel groups (placebo/antiemetic), a power of 80% and alpha (one-sided) 2.5% for Fisher's exact test. Note, however, that such a trial will of course be underpowered for smaller effects that might be of some clinical relevance in the absence of other evidence or in case the true incidence of recurrent PONV is lower than predicted.

Instead of using a placebo comparator researchers may think about using an active comparator and to compare the effectiveness of these two verum groups with each other or against a virtual margin based on the calculations presented here. Note, however, that such a proceeding comes at the price of not having demonstrated efficacy of any of the treatments compared to placebo. This approach addresses the special problem of placebo-control groups within this special niche of research and makes use of "historical" data of otherwise small trials that was pooled using meta-analysis. Despite all legitimate criticisms not to perform clinical trials without a placebo comparator, there are some issues within this special area of research that may justify the use of "historical" data. For the great majority of antiemetic drugs efficacy and effectiveness has been demonstrated in numerous prophylaxis trials with PONV setting and other indications where nausea and vomiting occur (e.g. radio- and chemotherapy for cancer). There is no biological rationale why antiemetics should not work postoperatively when their activity has been proven intraoperatively. Furthermore, meta-analyses that have been performed on the (few) placebo-controlled treatment studies available have yielded results very similar to the numerous meta-analyses that have been performed on the prophylaxis trials. Combined with the difficulties related to placebo control groups within this field and the ethical concerns of using placebo, one may justify the use of empirical rate of recurrent PONV after placebo or placebo/no treatment respectively for a single-arm study planning. By summarizing the existing data from an extensive literature search we can be confident that PONV re-occurs in at least 50% of the cases within the next 1–2 hours after a first event. Within the first 6 hours after a first episode PONV occurs in about at least 65% with no obvious further major increase (to 70–75%) when the 24-hour observation interval is used instead.

Since these "best-case" scenario statements are based on the lower 95% confidence interval bounds of the recurrent PONV rates, as a suggestion, any rate under antiemetic treatment should be better than these margins. This could mean that a similar 95%-confidence interval for the recurrent PONV rate under an investigational antiemetic intervention will not include such a "best-case" scenario value.

Tramèr and colleagues argue in favour of the use of placebo-controlled PONV-trials. They performed a systematic review of efficacy trials investigating ondansetron for the prophylaxis of PONV and found an enormous heterogeneity of the chance to develop nausea and vomiting (control event rate). In part this heterogeneity was assumed to be due to the random variation in small trials. We also observed between-trial variability for the recurrence of PONV. A closer look at figure 1 (PONV), however, suggests that very few trials are responsible for the variability. One trial comprises data from a Japanese group whose results have been repeatedly questioned for validity in the past [24,25]. However, since this study contributes only 20 patients it has no relevant impact on the global result of the analysis (e.g. estimate of PONV-recurrence after 24 hours observation period is 84% [95%-CI: 73%...91%] when the data of Fujii are included and 86% [95%-CI: 76%...92%] when they are removed from the meta-analysis). In the case of recurrent vomiting, again the report of Fujii [26] and a second small trial with 12 patients reporting on the effectiveness of small doses of propofol after gynaecological laparoscopy [27] might be viewed as outlier.

Besides the limited between-study heterogeneity, the inclusion of numerous small trials does not seem to increase the between-heterogeneity which is in contrast to other quantitative systematic reviews. In addition, the absence of an obvious bias induced by a high or low Jadad-score underlines the consistency of the analysis. The same holds true for the inclusion or exclusion of the data of the two observational studies where no placebo was administered but simply "no treatment" was given to the patients. Inclusion of both trials had a minor effect on the estimates (see table 2). Finally, in agreement with the expectations and as such another indicator of consistency the rate estimates increase with enlarged follow-up periods with PONV rate estimates being always at least as frequent as the estimates for vomiting.

## Conclusion

The great majority of trials that investigated the rate of recurrence of emetic symptoms after a placebo or placebo/no treatment within the early postoperative period demonstrate a consistently high incidence of emetic sequelae. Conducting a systematic search of the literature and statistical pooling of the available data using meta-analytic techniques, more reliable estimate of these incidences of recurrent symptoms can be determined. These numbers can be used for defining a first guess clinically relevant goal for future placebo controlled trials.

For several reasons, however, it is unlikely that randomized controlled trials will be performed that help to fill the gap between the huge knowledge that has accumulated by a plethora of antiemetic trials investigating the prophylaxis and the little knowledge on treatment of established symptoms due to the paucity trials on the treatment of PONV.

We conclude that the given estimates provide a solid approximation to "real life" and can be used with some confidence as a clue for future studies for instance in order to perform power calculations. As a rule-of-thumb, for the outcome "PONV" a recurrence rate of 65% can be used during a early period 0–2 hours after administering a placebo and 80% for a longer observation interval (0–6 hours or 0–24 hours respectively). For the recurrence of vomiting the expected incidences are 65% for the early period and 70–75% for the extended interval.

Using the lower 95%-confidence intervals of these estimates ("best-case" scenario) might be used as an indicator to guide future study planning. Within this special area of research, our data might even be used to come up with an alternative to a placebo controlled study design. Thus, research on antiemetic treatment which is urgently required might be stimulated.

## Competing interests

The author(s) declare that they have no competing interests.

## Authors' contributions

LE, HW, and PK performed the conceptual design of the study and drafted the manuscript. SF created the search algorithms of the systematic literature search and performed the search. HL and AM extracted and critically appraised the data. AS performed the statistical analysis and reviewed the draft version of the manuscript with respect to methodological issues.

All authors read and approved the final manuscript.

## Pre-publication history

The pre-publication history for this paper can be accessed here:


